# Associations of obstructive sleep apnea with truncal skeletal muscle mass and density

**DOI:** 10.1038/s41598-018-24750-z

**Published:** 2018-04-25

**Authors:** Takeshi Matsumoto, Kiminobu Tanizawa, Ryo Tachikawa, Kimihiko Murase, Takuma Minami, Morito Inouchi, Tomohiro Handa, Toru Oga, Toyohiro Hirai, Kazuo Chin

**Affiliations:** 1 Department of Respiratory Medicine, Graduate School of Medicine, Kyoto University, Kyoto, Japan; 2 Department of Respiratory Care and Sleep Control Medicine, Graduate School of Medicine, Kyoto University, Kyoto, Japan

## Abstract

Sarcopenia has been associated with several conditions relevant to obstructive sleep apnea (OSA), such as aging and obesity, but a direct relationship between OSA and skeletal muscle alterations has not been identified. This study investigated associations between computed tomography (CT)-measured skeletal muscle indices and OSA severity. Analyzed were 334 patients who underwent polysomnography to diagnose OSA. Lumbar skeletal muscles were assessed with CT for the skeletal muscle mass index (SMI, cross-sectional area, normalized for height squared) and skeletal muscle density (SMD, fat infiltration). The apnea-hypopnea index (AHI) correlated positively with the SMI and negatively with SMD in both men and women. The AHI was weakly associated with SMI only in men (β = 0.11, *P* = 0.017) after adjustment for the body mass index (BMI) (BMI: β = 0.61, *P* < 0.001 in men, β = 0.65, *P* < 0.001 in women). The association of AHI and SMD was not significant after adjustment for BMI (BMI: β = −0.42, *P* < 0.001 in men, β = −0.64, *P* < 0.001 in women). Severity of OSA correlated with increases in skeletal muscle mass rather than muscle depletion and skeletal muscle adiposity. These associations were limited compared with the stronger associations between obesity and skeletal muscles.

## Introduction

Sarcopenia is the loss of systemic skeletal muscle mass accompanied by decreased strength and function^1^. Sarcopenia has been of increasing interest because skeletal muscle depletion has been observed in several conditions such as aging, chronic obstructive pulmonary disease (COPD), metabolic syndrome, and cancer^2–6^. In fact, decreased skeletal muscle mass assessed by computed tomography (CT) and other measures was a significant predictor of overall survival of patients with cancer and COPD^4,7,8^. These findings suggest that skeletal muscle changes can reflect the systemic effects and burdens of chronic diseases. In addition, low skeletal muscle density (SMD) values on CT indicated fat infiltration into muscles and was associated with muscle dysfunction, aging, and obesity^2,3,9,10^. Thus, these CT measurements (mass volume and density) can be useful for assessment of different changes in skeletal muscles (depletion and fat accumulation) and could be associated with various conditions and their consequences.

Obstructive sleep apnea (OSA) is a common sleep disordered breathing condition. OSA has significant effects on various organ systems such as the cardiovascular (hypertension; ischemic heart disease), endocrinal (glucose intolerance; dyslipidemia), and central and sympathetic nervous systems (cognitive impairment; sympathetic activation)^11^. Among these comorbidities associated with OSA, hypertension, and diabetes are also associated with sarcopenia^12^. Given the wide range of the systemic impact of OSA, OSA can also profoundly affect skeletal muscles in the whole body. In addition, OSA was associated with ageing and obesity^13^, both of which were reported to be accompanied by skeletal muscle depletion and fat accumulation on CT measurements^2,3^. These findings suggest a possible association between OSA and skeletal muscles in the entire body. However, the direct relationship between OSA and skeletal muscle indices has not been investigated with the exception of one study that used the bioimpedance method^14^.

Thus, in this study, we aimed to investigate the relationship between OSA severity and skeletal muscle mass and density. We hypothesized that more severe OSA should be associated with greater losses of total skeletal muscle mass and increases in fat accumulation in muscles. CT and magnetic resonance imaging (MRI) are gold standards for estimating skeletal muscle mass^3^, and CT measurements at the lumbar level have been used as a surrogate marker for whole body skeletal muscle volume^7,15,16^. Therefore, using abdominal CT images taken to evaluate visceral fat accumulation and to diagnose visceral fat obesity, we assessed lumbar skeletal muscles quantitatively in patients suspected to have OSA as the representatives of total skeletal muscles. We then analyzed the associations among CT indices of skeletal muscles, OSA parameters, and other confounders such as body adiposity and self-reported physical activity.

## Results

### Baseline characteristics of study patients

**Figure 1:**
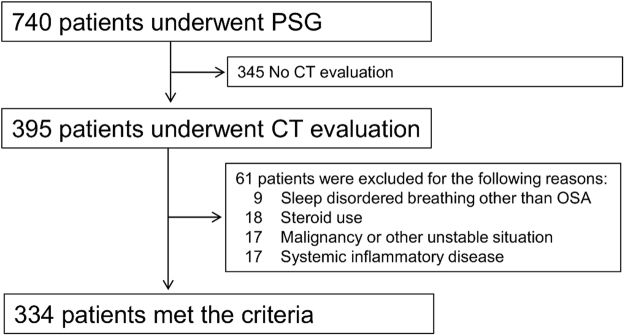
Flowchart of patient participation. PSG, polysomnography; CT, computed tomography; OSA, obstructive sleep apnea.

During the study period, 740 patients underwent polysomnography (PSG), and finally data on 334 patients were analyzed (Fig. 1). Of 334 patients included in the analysis, 12 (3.6%), 61 (18.3%), 105 (31.4%), and 156 (46.7%) had no (AHI < 5), mild (5 ≤ AHI < 15), moderate (15 ≤ AHI < 30), and severe OSA (AHI ≥ 30), respectively. Table 1 shows the baseline characteristics and skeletal muscle parameters of study patients according to sex. This comparison revealed that OSA was significantly more severe in the men than in the women, and the skeletal muscle mass index (SMI), SMD and subcutaneous fat area (SFA) index were significantly higher in the men than in the women (SMI, 52.5 ± 8.4 vs. 42.6 ± 7.5 cm^2^/m^2^; SMD, 35.8 ± 6.5 vs. 30.0 ± 7.8 cm^2^/m^2^; SFA index, 47.6 ± 22.1 vs. 76.6 ± 43.5 cm^2^/m^2^, all *P* < 0.001). Neither the BMI nor visceral fat area (VFA) index differed between the men and women.

**Table 1:** Patient characteristics and skeletal muscle parameters according to sex.

	All (n = 334)	Men (n = 239)	Women (n = 95)	P value
Age (years)	61.9 ± 10.4	61.8 ± 11.1	62.0 ± 10.6	0.875
BMI (kg/m^2^)	26.0 ± 4.7	25.9 ± 3.9	26.3 ± 6.4	0.560
AHI (events/h)	31.8 ± 20.0	34.8 ± 19.3	24.2 ± 19.6	<0.001
OSA severity, no/mild/moderate/severe (number, %)	12/61/105/156 (4/18/31/47)	3/33/73/130 (1/14/31/54)	9/28/32/26 (10/30/34/27)	<0.001
CT90^*^ (%)	4.6 [0.6–13.4]	6.8 [1.0–15.8]	1.5 [0.2–7.5]	<0.001
3%ODI (events/h)	28.8 ± 21.0	31.8 ± 20.7	21.4 ± 20.0	<0.001
Physical activity index^*,†^ (kcal/w)	857 [387–1825]	829 [371–1768]	942 [426–1986]	0.429
Maximum intensity^†^, no activity/light/moderate/vigorous (number, %)	29/54/134/44 (11/21/51/17)	21/36/91/36 (11/20/49/20)	8/18/43/8 (10/23/56/10)	0.311
Lumbar skeletal muscle area (cm^2^)	134.9 ± 33.1	148.4 ± 26.9	100.8 ± 20.0	<0.001
Skeletal muscle mass index (cm^2^/m^2^)	49.7 ± 9.3	52.5 ± 8.4	42.6 ± 7.5	<0.001
Skeletal muscle density (HU)	34.1 ± 7.4	35.8 ± 6.5	30.0 ± 7.8	<0.001
Visceral fat area (cm^2^)	115.7 ± 64.8	121.9 ± 64.0	100.2 ± 64.5	0.006
Visceral fat area index (cm^2^/m^2^)	43.0 ± 24.2	43.4 ± 23.1	42.2 ± 27.0	0.707
Subcutaneous fat area (cm^2^)	148.3 ± 81.5	134.9 ± 64.5	182.1 ± 106.7	<0.001
Subcutaneous fat area index (cm^2^/m^2^)	55.9 ± 32.5	47.6 ± 22.1	76.6 ± 43.5	<0.001

Abbreviations: BMI, body mass index; AHI, apnea-hypopnea index; CT90, cumulative percentage of sleep time with SpO_2_ < 90%; ODI, oxygen desaturation index; HU, Hounsfield Unit.

Data are expressed as means ± SD, numbers, percentages, or medians [interquartile range].

The severity of OSA was defined by AHI levels as follows: no, <5 events/h; mild, 5-<15 events/h; moderate, 15-<30 events/h; and severe, ≥30 events/h.

*Mann-Whitney U test was used, and the other continuous variables were calculated by unpaired Student’s t test.

^†^Data were collected for 261 patients (184 men, 77 women).

### Relationships between SMI and OSA, physical activity, and obesity

**Figure 2:**
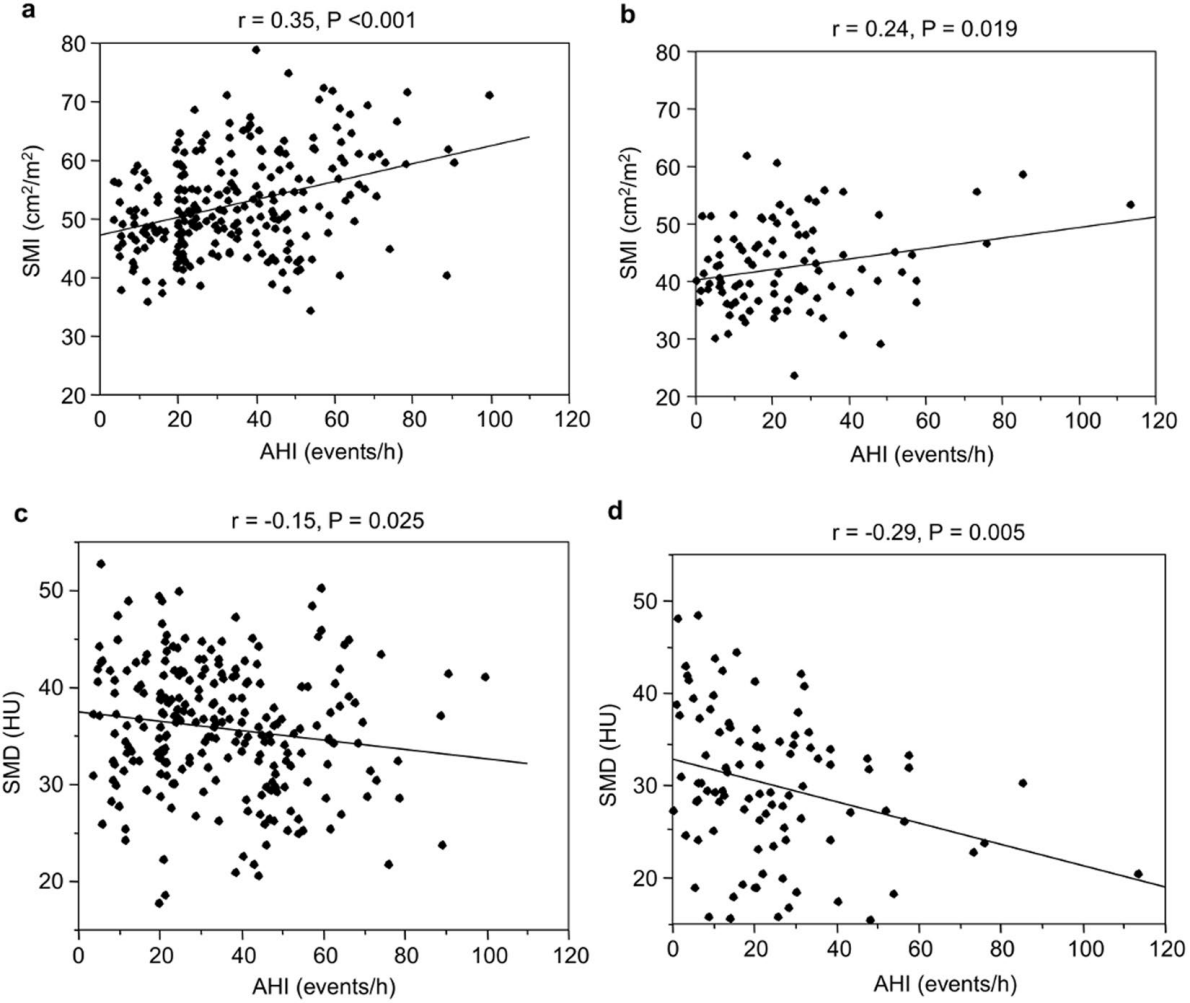
Correlation between AHI and the SMI or SMD. (**a**) Correlation between AHI and SMI in men. (**b**) Correlation between AHI and SMI in women. (**c**) Correlation between AHI and SMD in men. (**d**) Correlation between AHI and SMD in women. AHI, apnea-hypopnea index; SMI, skeletal muscle mass index; SMD, skeletal muscle density.

Given the significant gender differences in OSA severity and the skeletal muscle parameters, the relationships between skeletal muscle parameters and other clinical parameters were analyzed in men and women separately, as reported in previous studies^4,7,15–19^. Correlations between the SMI and clinical variables are shown in Table 2. The SMI was significantly correlated with AHI in both men and women (r = 0.35, *P* < 0.001, and r = 0.24, *P* = 0.019, respectively) (Fig. 2a,b).

**Table 2:** Correlation between skeletal muscle mass index or skeletal muscle density and clinical variables.

	Skeletal muscle mass index				Skeletal muscle density			
	Men		Women		Men		Women	
	r	P value	r	P value	r	P value	r	P value
Age (years)	−0.40	<0.001	−0.43	<0.001	−0.43	<0.001	−0.19	0.064
BMI (kg/m^2^)	0.72	<0.001	0.68	<0.001	−0.27	<0.001	−0.45	<0.001
AHI (events/h)	0.35	<0.001	0.24	0.019	−0.15	0.025	−0.29	0.005
CT90^*^ (%)	0.22	<0.001	0.12	0.233	−0.20	0.002	−0.37	<0.001
3%ODI (events/h)	0.36	<0.001	0.23	0.022	−0.15	0.023	−0.33	0.001
Physical activity index^*,†^ (kcal/w)	0.042	0.574	0.007	0.952	−0.12	0.120	−0.12	0.289
Visceral fat area index (cm^2^/m^2^)	0.40	<0.001	0.42	<0.001	−0.35	<0.001	−0.49	<0.001
Subcutaneous fat area index (cm^2^/m^2^)	0.50	<0.001	0.56	<0.001	−0.17	0.007	−0.43	<0.001

Abbreviations: BMI, body mass index; AHI, apnea-hypopnea index; CT90, cumulative percentage of sleep time with SpO_2_ <90%; ODI, oxygen desaturation index.

*Correlations between these variables and skeletal muscle mass index or skeletal muscle density were presented by Spearman rank correlation coefficients (ρ), and the others were presented by Pearson rank correlation coefficients.

^†^Data were analyzed for 261 patients (184 men, 77 women).

Results of the multivariate regression analysis of predictors for the SMI are shown in Table 3. Because there was strong collinearity between the BMI and the SFA index in men and women and between the BMI and VFA index in women, BMI (Table 3, model 1) and both the VFA index and SFA index (Table 3, model 2) were entered into the model separately. After adjusting for age and BMI, the AHI was significantly positively related to SMI in men (β = 0.11, *P* = 0.017), but the BMI was more strongly associated with the SMI in both sexes (Table 3, model 1). Fat mass was also associated with the SMI (Table 3, model 2). Even when we included the physical activity index as a covariate, the results were almost the same (Table 3, model 3). When CT90 or 3%ODI was chosen as a parameter of OSA severity instead of the AHI, 3%ODI was significantly positively related to the SMI in men (β = 0.12, *P* = 0.014), but CT90 was not (Supplementary Table S1).

**Table 3:** Multivariate regression analysis predicting skeletal muscle mass index in men and women.

	β	P value	R^2^	β	P value	R^2^
**Model 1**	**Men (n = 239)**			**Women (n = 95)**		
AHI (events/h)	0.11	0.017	0.040	—	0.379	
Age (years)	−0.23	<0.001	0.093	—	0.052	
BMI (kg/m^2^)	0.61	<0.001	0.437	0.65	<0.001	0.438
Cumulative R^2^			0.570			0.438
**Model 2**						
AHI (events/h)	0.17	0.003	0.061	—	0.759	
Age (years)	−0.32	<0.001	0.131	−0.28	0.003	0.121
Visceral fat area index (cm^2^/m^2^)	0.22	<0.001	0.087		0.204	
Subcutaneous fat area index (cm^2^/m^2^)	0.24	<0.001	0.118	0.34	0.013	0.190
Cumulative R^2^			0.397			0.311
**Model 3**	**Men (n = 184)**			**Women (n = 77)**		
AHI (events/h)	0.14	0.007	0.050	—	0.146	
Age (years)	−0.16	0.002	0.065	—	0.087	
BMI (kg/m^2^)	0.65	<0.001	0.466	0.72	<0.001	0.490
Physical activity index (kcal/w)	—	0.890		—	0.427	
Cumulative R^2^			0.581			0.490

Abbreviations: BMI, body mass index; AHI, apnea-hypopnea index; β, standardized regression coefficient; R^2^, coefficient of determination.

### Relationships between SMD and OSA, physical activity, and obesity

Table 2 shows the correlations between SMD and clinical variables. The SMD was significantly correlated with the AHI in both men and women (r = −0.15, *P* = 0.025, and r = −0.29, *P* = 0.005, respectively) (Fig. 2c,d).

Results of the multivariate regression analysis of predictors for SMD are shown in Table 4. As with the SMI, BMI (Table 4, model 1) and both the VFA index and SFA index (Table 4, model 2) were entered into the model separately. After adjusting for covariates, the AHI was not related to SMD in either model. However, age, BMI, VFA index, and SFA index were independently associated with SMD in both sexes. When CT90 or 3%ODI was chosen as a parameter of OSA severity instead of the AHI, neither CT90 nor 3%ODI was related to SMD (Supplementary Table S2).

**Table 4:** Multivariate regression analysis predicting skeletal muscle density in men and women.

	β	P value	R^2^	β	P value	R^2^
**Model 1**	**Men (n = 239)**			**Women (n = 95)**		
AHI (events/h)	—	0.939		—	0.823	
Age (years)	−0.55	<0.001	0.235	−0.45	<0.001	0.086
BMI (kg/m^2^)	−0.42	<0.001	0.116	−0.64	<0.001	0.290
Cumulative R^2^			0.351			0.376
**Model 2**						
AHI (events/h)	—	0.839		—	0.788	
Age (years)	−0.49	<0.001	0.211	−0.36	<0.001	0.069
Visceral fat area index (cm^2^/m^2^)	−0.26	<0.001	0.093	−0.27	0.026	0.131
Subcutaneous fat area index (cm^2^/m^2^)	−0.20	0.002	0.036	−0.38	0.007	0.162
Cumulative R^2^			0.340			0.362

Abbreviations: BMI, body mass index; AHI, apnea-hypopnea index; β, standardized regression coefficient; R^2^, coefficient of determination.

## Discussion

To the best of our knowledge, this is the first study to investigate the relationship between OSA severity and skeletal muscle mass and density as assessed by CT. In a relatively large cohort of patients suspected to have OSA, the AHI significantly and positively correlated with the SMI in both men and women. After adjustment for covariates, the association of the AHI with the SMI remained significant but was much weaker than that of the BMI in men; however, the association was not significant for women. The AHI was significantly and negatively correlated with SMD in men and women, but this association did not persist in either men or women after adjustment for other confounders.

We hypothesized that the severity of OSA negatively correlates with the SMI. However, our results were contrary to this hypothesis, showing that OSA was associated with an increased skeletal muscles mass in both men and women. Previously, the BMI was reported to be positively correlated with AHI^20^ and SMI^7^. Results of our multivariate analysis suggested that BMI was the strongest contributor to the SMI in our cohort. On the other hand, the independent association of OSA with the SMI was limited although statistically significant after adjustment for the BMI and age. This limited association of OSA with the SMI remained the same even when considering the effect of daily exercise. Similar results were obtained when VFA and SFA indices for the severity of obesity were entered into the analysis instead of the BMI. Taken together our findings suggested that skeletal muscle mass is mainly a reflection of body size and the severity of obesity in OSA patients.

The positive correlation between OSA severity and the SMI was contrary to the negative correlation observed with other chronic diseases such as cancer, chronic heart failure, and COPD. In those diseases, skeletal muscle depletion has been associated with the disease burden, probably through cachexia or systemic exhaustion caused by the disease. In lung, gastrointestinal, or skin cancer, decreased skeletal muscle mass was associated with impaired physical function and poor survival^7,21^. In chronic heart failure, skeletal muscle depletion has been related to reduced exercise capacity and the advancement of the disease^22^. In COPD, decreased skeletal muscle mass was associated with spirometric measurements indicating impaired lung function and poor survival^8,23^. Compared to these exhaustive diseases, OSA may have only a small role in skeletal muscle alterations, and the BMI and obesity may have much stronger relationships with the condition of skeletal muscles.

The severity of OSA was negatively correlated with SMD, suggesting that more severe OSA was associated with greater fat accumulation in muscles. This finding was consistent with our hypothesis, although the association was not independent from obesity. Obesity was shown to be a major confounder in the relationship between OSA and other conditions^24^. OSA was also reported to be associated with liver and visceral fat accumulation^25,26^, although the associations were much weaker than those between BMI and adiposity. Similarly, our results indicated that the relationship between OSA and adipose accumulation in skeletal muscles might be largely mediated by obesity.

Interestingly, some associations of OSA with skeletal muscle indices were different between men and women. For women, age and obesity (BMI and body fat indices) could account for all of these associations, while the AHI was independently associated with skeletal muscle mass only in men. Associations of OSA with BMI and adiposity were reported to differ between men and women. Women with OSA had a higher BMI than men with OSA of the same severity, suggesting that the impact of obesity could be greater in women^27^. The association of OSA with abdominal fat accumulation was stronger in men than in women, while obesity had a stronger association with abdominal fat in women^26,28^. These previous findings may be consistent with our results on skeletal muscle because obesity, rather than OSA, had a stronger link with adiposity in women than in men, although the mechanisms of sex differences relevant to OSA have not been elucidated.

Several limitations of this study should be mentioned. First, this was a cross-sectional study of an Asian population. Thus, because of its cross-sectional design our study could not reveal the causative relationship between skeletal muscle changes and OSA. In addition, we did not have CT measurements after continuous positive airway pressure (CPAP) therapy. A prospective longitudinal study of patients on CPAP therapy may clarify the impact of OSA on skeletal mass. Also, anthropometric values differ among ethnic groups^13,29^. Ethnic cohorts other than Asians should be studied. Second, skeletal muscles were assessed only in the lumbar region. A significant correlation between lumbar muscle mass measured by CT and MRI and total skeletal muscle volume assessed by dual-energy X-ray absorptiometry and MRI was reported^15,30^. Several studies evaluated skeletal muscles using CT images at the lumbar level as a surrogate marker for total skeletal muscle volume^7,16,18,31,32^. In addition, we showed a strong correlation between CT-measured skeletal muscle mass at the third lumbar vertebra and bioelectrical impedance analysis (BIA)-measured skeletal muscle mass of the whole body (Supplementary Figure S1). On the other hand, upper airway dilator muscle dysfunction has a specific role in the pathogenesis of OSA^33^. Hypoglossal nerve stimulation to dilator muscles has been proposed as a novel therapeutic option for OSA^34^. Thus, upper respiratory muscle changes may have different associations with OSA from other skeletal muscle changes, although we do not have data on upper airway muscles for analysis. The relationships between upper respiratory muscle changes and OSA should be addressed in future studies. Third, physical activity was assessed by questionnaires in this study, the results of which might be different from those obtained by objective measurements.

In conclusion, the severity of OSA did not correlate with skeletal muscle depletion. Conversely, patients with more severe OSA had a larger mass of skeletal muscles than those with less severe OSA even after adjustment for the strong effect of obesity, although this association was weak and limited to men. The severity of OSA was associated with low SMD, suggesting fat infiltration into skeletal muscles; however, this relationship was not independent from obesity. To clarify the roles of skeletal muscles in the pathogenesis of OSA and its consequences, these limited associations between OSA and skeletal muscle indices should be re-examined in future studies of other cohorts.

## Methods

### Patients

We retrospectively examined data on consecutive patients who underwent diagnostic full overnight PSG at the Sleep Unit of Kyoto University Hospital between January 2010 and December 2012. All had been referred to our sleep unit under suspicion of OSA with symptoms such as daytime sleepiness or habitual snoring. Inclusion criteria were age between 40 and 85 years, and no prior diagnosis of or treatment for OSA. Exclusion criteria were as follows: no CT scan, sleep disordered breathing other than OSA, steroid use, systemic inflammatory disease (i.e., connective tissue diseases), and malignancy. The Kyoto University Graduate School and Faculty of Medicine Ethics Committee approved this study (R0480) and waived the requirement for written informed consent from participants due to the study’s retrospective nature. All the performances were in accordance to the Declaration of Helsinki. Patient information was anonymized and de-identified prior to analysis.

### Polysomnography

The diagnosis of OSA was confirmed by overnight PSG (SomnoStar pro, Cardinal Health, Dublin, OH, USA or Alice 4, Philips Respironics, Inc., Murrysville, PA, USA) as previously described^35^. Apnea was defined as the continuous cessation of airflow for at least 10 s and hypopnea was defined as a reduction in airflow by nasal pressure ≥50% lasting at least 10 s accompanied by a decrease in SpO_2_ ≥3% and/or associated with arousal. Apnea-hypopnea index (AHI) values were calculated as the number of episodes of apnea and hypopnea per h over total sleep time.

### Physical activity

We assessed self-reported physical activity with questionnaires on frequency and time spent on three levels of physical activity. Three levels were vigorous (jogging, swimming, strenuous sports, bicycling on hills), moderate (housework, ballroom dancing, golf, bicycling on level ground, regular walking), and light (office work, strolling, personal care)^36^. Average intensity of activity was defined using metabolic equivalents (MET, kcal·kg^−1^·body weight·h^−1^) as follows: 9 METs for vigorous, 6 for moderate, and 3 for light^37^. The amount of physical activity (kcal/w) was calculated by multiplying the corresponding MET values by time spent and body weight, and summing up the values. The summed physical activity values were defined as the physical activity index. Also, the maximum intensity of physical activity (vigorous, moderate, light, or no activity) was used as the parameter of physical activity.

### Measurement of skeletal muscle and visceral and subcutaneous adipose tissue

Patients who agreed to our recommendation underwent an abdominal CT within 30 days of the diagnostic PSG before starting CPAP therapy. Unenhanced transverse CT was performed with an Aquilion 64 CT system (Toshiba Medical Systems Corporation, Tochigi, Japan). In this study, CT images of 7 mm slices were used for the analysis of skeletal muscle area and muscle density.

For skeletal muscle, two consecutive transverse CT images extending from the third lumbar vertebrae in the inferior direction were assessed, then averaged. Skeletal muscle tissues quantified included the psoas, erector spinae, quadratus lumborum, transversus abdominus, internal and external obliques, and rectus abdominus. Images were analyzed with a workstation (AZE VirtualPlace 99, AZE of America, Ltd., Irvine, CA, USA). Skeletal muscle was identified and quantified by Hounsfield Unit thresholds of −29 to +150. Cross-sectional area for muscle was normalized for height in meters squared (cm^2^/m^2^) and described as the SMI^4,7,15,16,18,19,31,32^. SMD, which was studied as a correlate of muscle density determined by CT^38^, was reported for the entire muscle area at the third lumbar vertebra^7,18^. The intraclass correlation coefficients for the SMI and SMD from 50 random subset samples were both >0.95.

The lumbar skeletal muscle area was shown to be strongly correlated with the whole skeletal muscle mass^15,30^. To determine whether SMI at the third lumbar vertebra is valid as a surrogate index for total skeletal muscle volume, we investigated the association between SMI and the whole body skeletal muscle mass obtained from BIA in another cohort of OSA patients (n = 68). For BIA, patients were assessed in the supine position by a body composition analyzer (InBody S20; Biospace, Seoul, Korea). Appendicular skeletal muscle mass (ASM) normalized for height in meters squared was used as an indicator of total skeletal muscle volume^39^. SMI was strongly correlated with ASM/height^2^ (r = 0.804, *P* < 0.001, Supplementary Figure S1).

For the VFA and SFA, CT images at the level of the umbilicus were assessed^40^. VFA and SFA were quantified with the same workstation mentioned above and as previously described^26^. VFA and SFA were both normalized for height in meters squared (cm^2^/m^2^) and described as the VFA index and SFA index, respectively.

### Statistical analysis

To evaluate the clinical features of each sex, we classified the participants into men and women. All values were expressed as means ± standard deviations, percentages, or medians [interquartile range] unless stated otherwise. Continuous variables were compared using an unpaired Student’s *t* test or Mann-Whitney U test, as appropriate. Categorical variables were compared using a chi-squared test or Fisher’s exact test, as appropriate. To analyze the correlation between the severity of OSA and the SMI or SMD, Pearson or Spearman rank correlation coefficients were calculated for each sex, as appropriate. To test our hypothesis that SMI and SMD were associated with the severity of OSA, we performed a multivariate regression analysis for each sex. As covariates for SMI, we included age, BMI, and the physical activity index, which were shown to be associated with muscle mass^4,7,15,16,19,32,41^, and the VFA index, SFA index, and AHI, the latter of which is usually taken as a parameter of OSA severity. As covariates for SMD, we included age, BMI, VFA index, and SFA index, which were associated with muscle density^7,17,18,38^, and AHI. When multiple independent variables had strong collinearity (r >0.70), one was selected. In the sample size calculation, when the effect size was 0.15, the statistical power level was 0.8, the number of predictors was 4, and the probability level was 0.05, the minimum required sample size for multiple regression analysis was 84. During 2010–2012, the number of eligible female patients was 95 and that of male patients was 239. Thus, we decided to include all consecutive patients during this period. Two-tailed P value < 0.05 was deemed statistically significant. All statistical analyses were performed using JMP 11.2.0 software (SAS Institute Inc., Cary, NC, USA).

### Data availability

The datasets analyzed during the current study are available from the corresponding author on reasonable request.

## Electronic supplementary material

Supplementary material
