# Supplementary material for: Associations of obstructive sleep apnea with truncal skeletal muscle mass and density
Source: Sci Rep. 2018 Apr 25;8:6550. doi: 10.1038/s41598-018-24750-z (PMC5916913; doi:10.1038/s41598-018-24750-z)
Supplement: Supplementary file 1 — Supplementary material [file 41598_2018_24750_MOESM1_ESM.pdf]

Supplementary material

Associations of obstructive sleep apnea with truncal skeletal muscle mass and density

Associations of obstructive sleep apnea with truncal skeletal muscle mass and density

Takeshi Matsumoto, Kiminobu Tanizawa, Ryo Tachikawa, Kimihiko Murase, Takuma Minami, Morito Inouchi, Tomohiro Handa, Toru Oga, Toyohiro Hirai, Kazuo Chin*

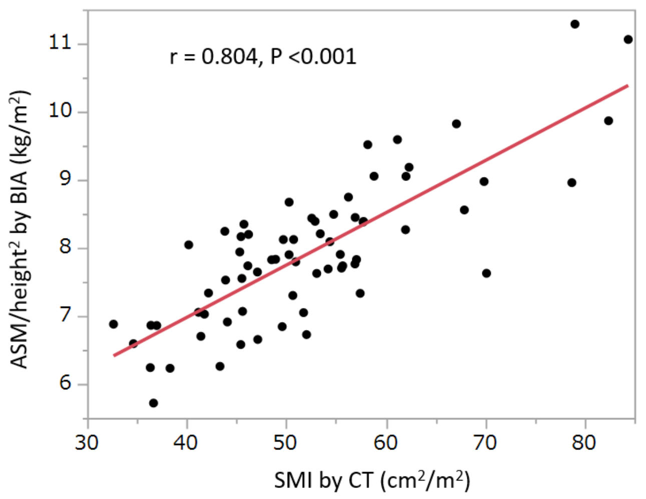

Supplementary Figure S1. Correlation between SMI and ASM normalized for height in meters squared by BIA.

SMI, skeletal muscle mass index; CT, computed tomography; ASM, appendicular skeletal muscle mass; BIA, bioelectrical impedance analysis.

Supplementary Table S1. Multivariate regression analysis of predicting skeletal muscle mass index using CT90 or 3%ODI instead of AHI

Model 1	Men (n = 239)			Women (n = 95)		
	β	P value	R^2	β	P value	R^2
CT90 (%)	-	0.725		-	0.180	
Age (years)	-0.22	<0.001	0.090	-0.17	0.037	0.074
BMI (kg/m^2)	0.65	<0.001	0.485	0.65	<0.001	0.441
Cumulative R^2			0.575			0.515
**Model 2**	**Men (n = 239)**			**Women (n = 95)**		
	β	P value	R^2	β	P value	R^2
3%ODI (events/h)	0.12	0.014	0.041	-	0.202	
Age (years)	-0.23	<0.001	0.094	-	0.078	
BMI (kg/m^2)	0.61	<0.001	0.435	0.68	<0.001	0.458
Cumulative R^2			0.570			0.458

(Model 1) Results using CT90 instead of AHI. (Model 2) Results using 3%ODI instead of AHI. Abbreviations: BMI, body mass index; CT90, cumulative percentage of sleep time with SpO_2 <90%; ODI, oxygen desaturation index; β, standardized regression coefficient; R^2, coefficient of determination.

Supplementary Table S2. Multivariate regression analysis of predicting skeletal muscle density using CT90 or 3%ODI instead of AHI

Model 1	Men (n = 239)			Women (n = 95)		
	β	P value	R^2	β	P value	R^2
CT90 (%)	-	0.455		-	0.649	
Age (years)	-0.54	<0.001	0.233	-0.44	<0.001	0.085
BMI (kg/m^2)	-0.41	<0.001	0.112	-0.61	<0.001	0.276
Cumulative R^2			0.345			0.361
Model 2	Men (n = 239)			Women (n = 95)		
	β	P value	R^2	β	P value	R^2
3%ODI (events/h)	-	0.898		-	0.897	
Age (years)	-0.55	<0.001	0.235	-0.45	<0.001	0.086
BMI (kg/m^2)	-0.43	<0.001	0.118	-0.64	<0.001	0.288
Cumulative R^2			0.353			0.374

(Model 1) Results using CT90 instead of AHI. (Model 2) Results using 3%ODI instead of AHI. Abbreviations: BMI, body mass index; CT90, cumulative percentage of sleep time with SpO_2 <90%; ODI, oxygen desaturation index; β, standardized regression coefficient; R^2, coefficient of determination.
